# 974. Real World Experience of Posaconazole Therapeutic Drug Monitoring in Oncology Patients: Clinical Implications of Hypoalbuminemia as a Predictor of Subtherapeutic Posaconazole Levels

**DOI:** 10.1093/ofid/ofad500.029

**Published:** 2023-11-27

**Authors:** Yanina Pasikhova, John Greene, Austin R Morrison, Guy Handley

**Affiliations:** Moffitt Cancer Center, Tampa, FL; Moffitt Cancer Center, Tampa, FL; Moffitt Cancer Center, Tampa, FL; University of South Florida, Tampa, Florida

## Abstract

**Background:**

Posaconazole (POS) is utilized for prophylaxis or treatment of invasive fungal infections (IFI) in patients with malignancies or following stem cell transplant (SCT). Subtherapeutic levels may be associated with breakthrough infection and therapeutic drug monitoring (TDM) is recommended. Timely measurements are not available at most institutions. We sought to identify factors associated with subtherapeutic POS levels in high-risk patients for IFI.

**Methods:**

Patients at an NCI designated cancer center with POS level from 06/2021 to 07/2022 were evaluated for risk factors associated with subtherapeutic levels. Only patients with at least one level receiving oral delayed release tablets or IV formulations were included.

**Results:**

A total of 66 patients were identified with 61 included. 46 had hematologic malignancies, 27 underwent SCT, 4 were treated with chimeric antigen receptor T cell therapy. Majority were male with a median age of 64 years old, a median BMI of 26.26 and a weight of 75.9 kilograms.

POS was used for treatment of IFI in 53 patients. Median time from initiation to level was 13 days. The median level was 1.74mcg/mL. whereas for patients with a BMI above 30 (17/61), median level was 1.17mcg/mL. Hypoalbuminemia, defined as ≤ 3g/dL, was seen in 28 patients and was associated with statistically higher rates of sub-therapeutic levels for target levels reported for treatment of 1.25mcg/mL (18/28; p=0.004), 1.5mcg/mL (20/28; p=0.012) and 1.8mcg/mL (22/28; p=0.016).

Only one breakthrough IFI occurred on patients receiving POS for prophylaxis compared to two patients receiving POS for treatment: both with subtherapeutic levels. Only one patient discontinued the drug due to intolerance. No discontinuations occurred due to liver toxicity or QTc prolongation.

**Conclusion:**

Therapeutic levels are critical for treatment and prophylaxis of IFI in oncology patients and delays in accurate measurements are common. In our analysis, hypoalbuminemia was associated with subtherapeutic levels at standard dosing, and higher initial dosing may be considered in high-risk patients with hypoalbuminemia particularly for treatment of IFI.

**Disclosures:**

**All Authors**: No reported disclosures

